# Inhibitory Impact of Prenatal Exposure to Nano-Polystyrene Particles on the MAP2K6/p38 MAPK Axis Inducing Embryonic Developmental Abnormalities in Mice

**DOI:** 10.3390/toxics12050370

**Published:** 2024-05-17

**Authors:** Junyi Lv, Qing He, Zixiang Yan, Yuan Xie, Yao Wu, Anqi Li, Yuqing Zhang, Jing Li, Zhenyao Huang

**Affiliations:** 1 Key Laboratory of Human Genetics and Environmental Medicine, School of Public Health, Xuzhou Medical University, Xuzhou 221004, China; 202005010319@stu.xzhmu.edu.cn (J.L.); 302110110929@stu.xzhmu.edu.cn (Q.H.); 202005010420@stu.xzhmu.edu.cn (Z.Y.); 202005010315@stu.xzhmu.edu.cn (Y.X.); 202105010105@stu.xzhmu.edu.cn (A.L.); 100002008046@xzhmu.edu.cn (J.L.); 2 School of Medical Imaging, Xuzhou Medical University, Xuzhou 221004, China; 3 Department of Obstetrics and Gynecology, Women’s Hospital of Nanjing Medical University, Nanjing Maternity and Child Health Care Hospital, Nanjing 210004, China; yuqingzhang@njmu.edu.cn

**Keywords:** nanoplastics, exposure during pregnancy, placenta

## Abstract

Nanoplastics, created by the fragmentation of larger plastic debris, are a serious pollutant posing substantial environmental and health risks. Here, we developed a polystyrene nanoparticle (PS-NP) exposure model during mice pregnancy to explore their effects on embryonic development. We found that exposure to 30 nm PS-NPs during pregnancy resulted in reduced mice placental weight and abnormal embryonic development. Subsequently, our transcriptomic dissection unveiled differential expression in 102 genes under PS-NP exposure and the p38 MAPK pathway emerged as being significantly altered in KEGG pathway mapping. Our findings also included a reduction in the thickness of the trophoblastic layer in the placenta, diminished cell invasion capabilities, and an over-abundance of immature red cells in the blood vessels of the mice. In addition, we validated our findings through the human trophoblastic cell line, HTR-8/SVneo (HTR). PS-NPs induced a drop in the vitality and migration capacities of HTR cells and suppressed the p38 MAPK signaling pathway. This research highlights the embryotoxic effects of nanoplastics on mice, while the verification results from the HTR cells suggest that there could also be certain impacts on the human trophoblast layer, indicating a need for further exploration in this area.

## 1. Introduction

**Figure 1:**
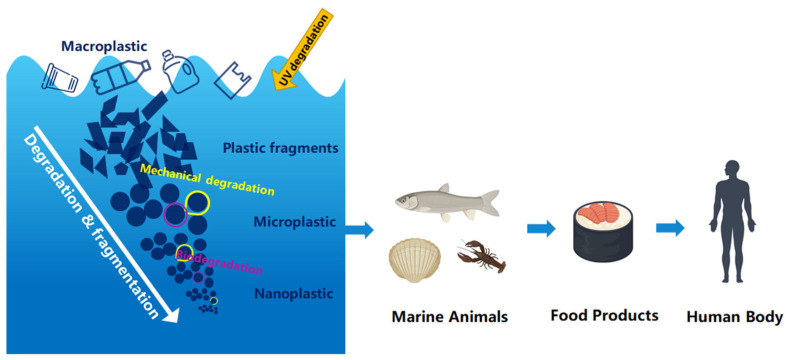
The sources, formation, and impact pathways of microplastics and nanoplastics on living organisms.

Nanoplastics are formed from the degradation of larger plastic debris, initially into microplastics and eventually into nanoparticles that are less than 100 nm in size (Figure 1) [1,2]. This process is instigated by environmental factors such as ultraviolet radiation, oxygen, and mechanical abrasion from waves and sand, and can be influenced by temperature and certain microorganisms [3]. Over time, these factors gradually break down microplastics into nanoplastics. The main types of nanoplastics include polymers such as polyethylene (PE), polypropylene (PP), polystyrene (PS), and polyvinyl chloride (PVC).

The pollution caused by nanoplastics is indeed severe and poses various environmental and health issues. Due to their small size, nanoplastics have the ability to pervade different environmental compartments, including water, air, and soil. Unfortunately, as it stands currently, specific data on the global scale of nanoplastics pollution are limited. This is largely because nanoplastics are difficult to detect, measure, and isolate, given how extremely small they are. Even the most cutting-edge scientific methods have difficulty measuring nanoplastics accurately. However, to give a sense of the broader scale of plastic pollution, a study estimated that 8.3 billion tons of plastic have been produced since the 1950s, and about 60% of that plastic has ended up in either a landfill or the natural environment [4]. Meanwhile, the number of microplastic particles in our oceans is a massive 51 trillion [5]. While these figures do not provide a direct measure of nanoplastics, they underscore the seriousness of the broader plastic pollution issue, and these plastics will eventually break down into microplastics and nanoplastics in the environment, posing a greater potential hazard to the environment [6].

Microplastics and nanoplastics can easily enter the food chain as they can be ingested by small organisms and subsequently reach larger ones, potentially impacting wildlife and human health (Figure 1). A 2019 World Wildlife Fund report reveals that globally, each person ingests an average of 2000 microplastic particles weekly, weighing the same as a credit card [7]. Scientists have estimated the volume of microplastics inhaled by humans within a 24 h period to be approximately 272 particles, according to air samples taken from three apartments and a constructed human model [8]. However, nanoplastics originate from the degradation of plastic fragments, and it is estimated that the concentration of these fragments is 10^14^ times higher than that of the microplastics currently found in the aquatic environment [9]. Nanoplastics enter the human body mainly through the respiratory tract, the digestive tract, and the skin, and they can produce toxic effects on the corresponding tissues and organs through particle internalization or migration. Nanoplastics have been reported to exert toxic effects on the digestive, neurological, respiratory, immune, and reproductive endocrine systems by physically damage, causing an imbalance in intestinal flora, altering enzyme activity, activating immune cells, inducing oxidative stress, and interfering with endogenous hormones [10,11]. In animal experimental studies, it was found that exposure to nanoplastics can lead to thickening of mouse alveolar walls and pulmonary interstitial fibrosis, causing changes in lung structure [12]. Concurrently, they also cause liver damage, characterized by the infiltration of immune cells, hepatocyte vacuolization, nuclear shrinkage, and enlargement of hepatic sinusoidal spaces. The damage to the kidneys is primarily manifested in the form of renal tubule and glomerular atrophy accompanied by an inflammatory response. Exposure to nanoplastics also causes neurotoxicity in zebrafish, leading to abnormal behavior in zebrafish [13]. Nanoplastics can also disrupt the composition and function of the zebrafish gut microbiota, accompanied by immune system dysfunction [14]. This disrupts the brain–gut axis, mediated by alterations in neurotransmitter metabolites. Moreover, nanoplastics can act as carriers for other pollutants, as they have a large surface area that can adsorb harmful substances such as heavy metals and persistent organic pollutants [15,16,17].When ingested by organisms, these substances can be released and cause further harm.

Embryonic development is a crucial stage in an organism’s life, during which the organism undergoes rapid growth and cell division. It is agreed upon in many scientific studies that the embryo is often more sensitive to various environmental stressors, including pollution, as compared to adults. Regarding nanoplastics, little is known about their specific effects on embryonic development. However, given that nanoplastics can have harmful effects on adult organisms [10], it is reasonable to hypothesize that embryos, being in a more vulnerable and sensitive stage, might be even more adversely affected by nanoplastics exposure. A few experimental studies on aquatic organisms have indicated that exposure to nanoplastics during the embryonic stage could cause developmental abnormalities [18]. Polystyrene nanoplastic particles (PS-NPs) reduced nutrient accumulation and led to inhibition of gonadal development in juvenile river prawns [19]. PS-NPs also induce neurotoxicity (decreased spontaneous contraction frequency), cardiotoxicity (bradycardia), and morphological changes in the eyes and head of zebrafish embryos, leading to impaired embryonic development [20,21]. In addition, population studies have found higher levels of nanoplastic particles in the chorionic tissue of patients, with unexplained recurrent miscarriage (RM) relative to healthy individuals [22]. However, more research is needed to conclusively determine the risks and understand how nanoplastics could affect embryos.

In this research, our primary objective was to unravel the potential toxicological mechanisms of polystyrene nanoparticles (PS-NPs) on ICR mice during pregnancy and human trophoblast cells. We constructed a model of PS-NP (30 nm) exposure during pregnancy in mice to explore the effects and specific mechanisms of PS-NP exposure during pregnancy on dams and offspring. Through this study, we aimed to validate our argument that a certain concentration of small-sized PS-NPs is toxic to embryonic development and to probe potential mechanisms responsible for this toxicity.

## 2. Materials and Methods

### 2.1. Nanoplastics and Characterization

Polystyrene nanoplastic particles (PS-NPs) were purchased from Rigor Science (Wuxi, China), with a diameter of 30 nm and a mass percentage of 2.5 wt%. The TEM images were obtained using a JEM-2000EX microscope (JEOL, Tokyo, Japan). The size and zeta potential of PS-NPs were determined using a Zetasizer Nano (ZS90, Worcester, UK). Each measurement was made three times at a controlled temperature of 25 ± 1 °C.

### 2.2. In Vivo Experiments

#### 2.2.1. Modeling of PS-NP Exposure during Pregnancy in ICR Mice

The study was conducted using 8-week-old ICR mice of the Specific Pathogen Free (SPF) category, provided by the Experimental Animal Center of Xuzhou Medical University, with ethical approval granted by the Xuzhou Medical University Ethics Committee (Ethical Approval Number: 202305T002). The ICR mice were kept in an SPF-grade animal room with controlled temperature conditions (22 ± 2 °C), and subjected to alternating 12 h light and dark cycles. They had unrestricted access to food and water. One week was allocated prior to the experiment for the mice to adjust to their surroundings.

Female and male mice were co-housed in a 2:1 ratio and vaginal plug checks were carried out each morning. The identification of a vaginal plug is a conventional sign used in reproductive biology to designate that mating has occurred in mice. The day a plug is observed is defined as gestational day 0.5 (GD0.5). The study used 30 nm diameter PS-NPs and divided the pregnant mice (*n* = 20) randomly into four groups for the experiment: control group (corn oil), low-dose group (0.1 mg/kg/d), medium-dose group (1 mg/kg/d), and high-dose group (10 mg/kg/d), comprising 5 mice each. Following pregnancy confirmation, gavage of PS-NP solution was conducted based on the body weight of the pregnant mice, while also documenting their food and water intake. This continued until GD18.5, when the pregnant mice were euthanized.

#### 2.2.2. Histopathology

Upon euthanizing the pregnant mice on GD18.5, organs such as the placenta and liver were harvested for subsequent pathological examination. Using tweezers, the placenta and fetuses were dissected, cleaned in physiological saline, weighed, and recorded. Five placentas per group (one placenta corresponding to each fetus) were then placed in a 4% paraformaldehyde solution to fix. After a process of gradient dehydration with ethanol, embedding, and sectioning, Hematoxylin and Eosin (H&E) staining was performed. Under the microscope, changes in the placental tissue pathology were observed.

#### 2.2.3. Placental Transcriptome Sequencing

The placentas collected from both the control group and the PS-NP exposure group were used for transcriptome sequencing (3:3); the specific steps were similar to those in previous studies and are briefly described below [23,24]. The transcriptome profiling protocol commenced with the isolation and purification of the total RNA from the placental tissues using TRIzol reagent (Invitrogen, Waltham, MA, USA) in accordance with the manufacturer’s instructions. Subsequently, cDNA libraries were prepared for next-generation sequencing using a NEBNext^®^ Ultra™ RNA Library Prep Kit for Illumina^®^ (New England Biolabs, Ipswich, MA, USA) following the manufacturer’s recommendations, which subsequently generated extensive transcriptome data. The raw data were initially processed through in-house Perl scripts and clean data were obtained. Thereafter, all downstream analyses were based on clean data with high quality. Q20, Q30, and GC contents of the raw data were calculated. We utilized the Majorbio Cloud Platform (https://cloud.majorbio.com/ (accessed on 05 January 2024)) to analyze the aligned reads. When the false discovery rate (FDR) < 0.01 and Fold Change ≥ 2, differentially expressed genes (DEGs) were identified between the control and PS-NP-exposed placentas. Finally, the classification and functional enrichment of the DEGs were analyzed using Kyoto Encyclopedia of Genes and Genomes (KEGG) databases and Gene Ontology (GO) databases. Multiple testing corrections were made using FDR and only those pathways having an adjusted *p*-value < 0.05 were considered to be significantly enriched.

### 2.3. In Vitro Experiments

#### 2.3.1. Cell Sources and Cultures

HTR-8/SVneo cells, abbreviated as HTR, were used for cell experiments (bought from HyCyte, Suzhou, China). HTR-8/SVneo cells were obtained by transfecting cells grown from human early gestation placental villous explants with a gene encoding the simian virus 40 large T antigen, and can be used to study trophoblast and placental biology [23,24]. The cells were cultured in RPMI-1640 Medium (P/S) (KeyGEN BioTECH, Nanjing, China) with added PS-NP solution and 10% fetal bovine serum (ZETA, Spring House, PA, USA). The concentrations of PS-NPs used were 0, 10, 20, 50, and 100 μg/mL.

#### 2.3.2. Cell Proliferation Assays

Cell proliferation vitality was evaluated using a CCK8 assay. The seeding density of HTR was 2.5 × 10^4^ mL^−1^. After 24 h, PS-NPs were added to the culture medium for another 24 h. As per the manufacturer’s instructions, a cell proliferation and cytotoxicity kit-8 (CCK8) was used (Abbkine Scientific, Wuhan, China). After incubation for 2 h, cell viability was determined under 450 nm absorbance with an enzyme-linked immunosorbent assay to assess the cell proliferation rate.

#### 2.3.3. Cell Migration Assay

The migration ability of HTR cells was evaluated using a cell scratch test. Fully grown HTR cells were evenly inoculated into a six-well plate. After incubation for 24 h, once the cells were fully merged, a scratch was made using a 20 μL sterile pipette tip. After washing with warm PBS to remove floating cells, images were taken at the 0 h mark. The PBS was then removed from the six-well plate, and PS-NPs were added to the fetal bovine serum containing the culture medium. According to the standard of 2 mL per well, this was added to the six-well plate for further cultivation. After 24 h, scratch images were taken at the same location. The scratch area was then measured using ImageJ 1.53e software (Wayne Rasband, Bethesda, MA, USA)

### 2.4. RNA Extraction and Reverse Transcription-Quantitative Polymerase Chain Reaction (qRT-PCR)

Total RNA was extracted from cells and tissues using TRIzol reagent Vazyme BioTech (Nanjing, China), following the manufacturer’s provided method. The total RNA was then quantitated using the nanodrop 2000c system (Thermo Fisher Scientific, Waltham, MA, USA). The RNA was then reverse transcribed into cDNA using HiScript II Q RT SuperMix for qPCR (+gDNA wiper) Vazyme BioTech (China), and amplified with ChamQ SYBR qPCR Master Mix (Applied Biosystems, Los Angeles, CA, USA) at a volume of 10 μL. Real-time fluorescence quantitative PCR was carried out based on the ChamQ SYBR qPCR Master Mix (Vazyme BioTech, China). The data were normalized using GAPDH. The primer sequences can be seen in the Supplementary Material, Table S1.

### 2.5. Western Blotting

After treating the cells with PS-NPs at concentrations of 0, 10, 20, 50, and 100 μg/mL for 24 h, the HTR cells were lysed with RIPA lysis buffer (Beyotime Biotechnology, Shanghai, China) containing proteinase and phosphatase inhibitors (Beyotime Biotechnology, China). Protein concentration was measured using a BCA reagent kit and proteins were denatured by adding SDS and PBS and heating at 100 °C for 5 min. After gel electrophoresis, the proteins were separated and transferred to a polyvinylidene fluoride (PVDF) membrane. QuickBlock Western Blocking Solution (Beyotime Biotechnology, China) was used for blocking for 20 min. After incubation with the primary antibody at 4 °C overnight, the membranes were washed with TBST (Servicebio, Wuhan, China), and then subjected to incubation with the secondary antibody for 1 h before being washed again with TBST. Finally, the PVDF membranes were imaged using a Bio-Rad ChemiDocXRS+ (Bio-Rad, Hercules, CA, USA). Protein band grayscale values on the images were calculated using Image J.

### 2.6. Statistical Analysis

The data collected from this experiment were analyzed using SPSS 21.0 statistical software. All data are represented as mean ± standard deviation. A test for homogeneity of variance was first conducted, followed by the *t*-test, Mann–Whitney U test, and Friedman test. Graph Pad Prism 5.0 was used for statistical plotting. A *p*-value below 0.05 indicates a statistically significant difference. The mechanism figure was drawn using Figdraw 2.0 (ResearchHome, Hangzhou, China).

## 3. Results

### 3.1. Characteristics of PS-NPs

The transmission electron microscope results revealed that the original particle size of PS-NPs was about 30 nm. They were in the form of spherical particles with good dispersion (Supplementary Material, Figure S1A). The hydrodynamic diameter of the PS-NPs was 167.7 ± 0.22 nm, larger than their original particle size, indicating a slight agglomeration of PS-NPs in water (Supplementary Material, Figure S1B). The zeta potential of the PS-NPs was −23.0 ± 5.0 mV (Supplementary Material, Figure S1C). At the same time, the Polymer Dispersion Index (PDI) of PS-NPs was less than 0.5, indicating good dispersion. These results demonstrate that PS-NPs possess common nanoparticle characteristics and meet the experimental requirements; thus, they could be used for subsequent experiments.

### 3.2. Embryonic Developmental Toxicity of PS-NPs in ICR Mice

Compared with the control group, there was no significant change in the body weight of pregnant mice fed with different doses of PS-NPs (Supplementary Material, Figure S2A). The body weight changes among all four pregnant mice groups (from GD0.5 to GD18.5) showed a steady increasing trend with no significant differences (Supplementary Material, Figure S2B). In addition, there was no difference in food and water intake during the exposure period between different dose groups and the control group (Supplementary Material, Figure S2C,D).

**Figure 2:**
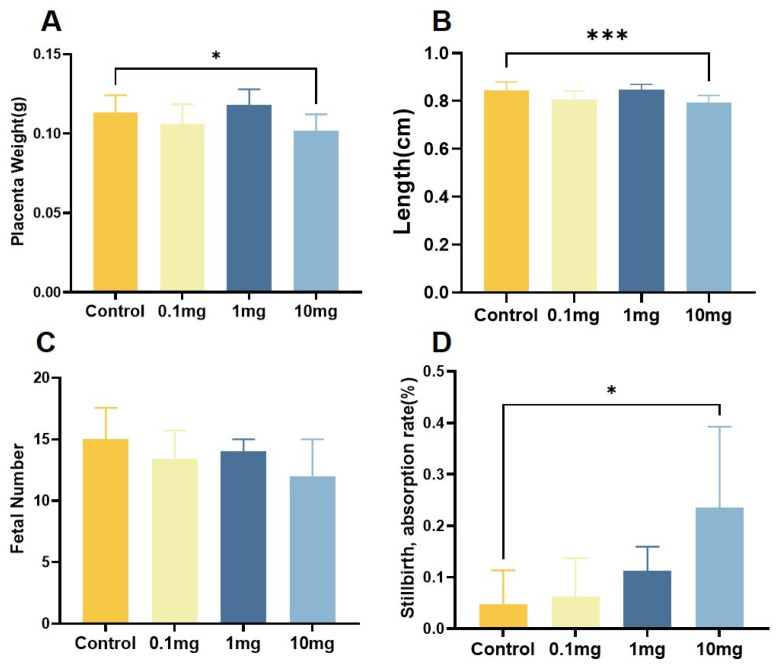
Biological effects of PS-NPs on fetal rats. (**A**) Placenta weight. (**B**) Placenta diameter. (**C**) Number of embryos. (**D**) Stillbirth, absorption rate. Data are represented as the mean ± SEM. * *p* < 0.05, *** *p* < 0.001.

**Figure 3:**
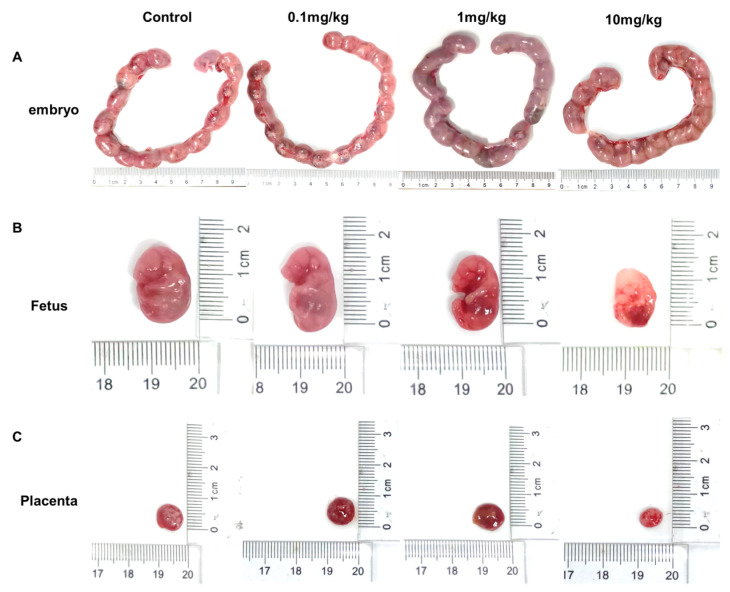
Exposure to PS-NPs causes placental and fetal developmental toxicity. (**A**) Representative images of maternal rat embryos. (**B**) Representative image of a fetal rat. (**C**) Representative image of placenta.

Compared with the control group, both the placental weight and diameter in the 10 mg/kg/d group decreased, although the placental weight and diameter in the 1 mg/kg/d group increased, and the differences were not statistically significant (Figure 2A,B and Figure 3). Meanwhile, the number of embryos in each exposure group showed no significant differences compared to in the control group (Figure 2C). At the same time, the high-dose group showed a significantly higher rate of fetal death and absorbed fetuses compared to the control group (Figure 2D).

**Figure 4:**
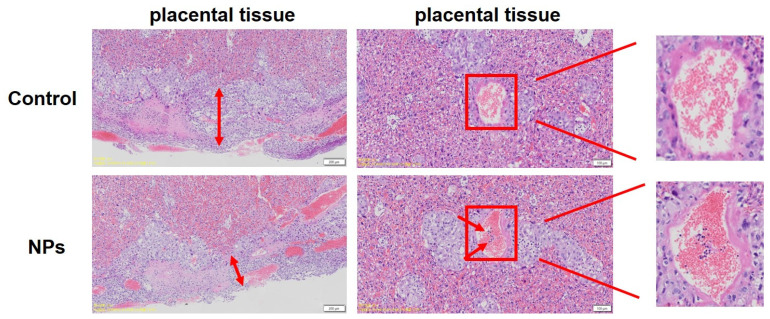
Exposure to PS-NPs resulted in pathological changes in the placenta at GD18.5.

By observing the morphological structures of the placenta via HE staining, it was found that in the PS-NP exposure group, the nourishing layer of the placenta became thinner, the invasion of nourishing cells was insufficient, and there was a large number of immature red blood cells in the blood vessels (Figure 4). All these results suggest that exposure to PS-NPs during pregnancy may lead to poor placental development, which could further cause embryonic development obstacles.

### 3.3. Screening of Differentially Expressed Genes in Placental Tissues Exposed to PS-NPs during Pregnancy

**Figure 5:**
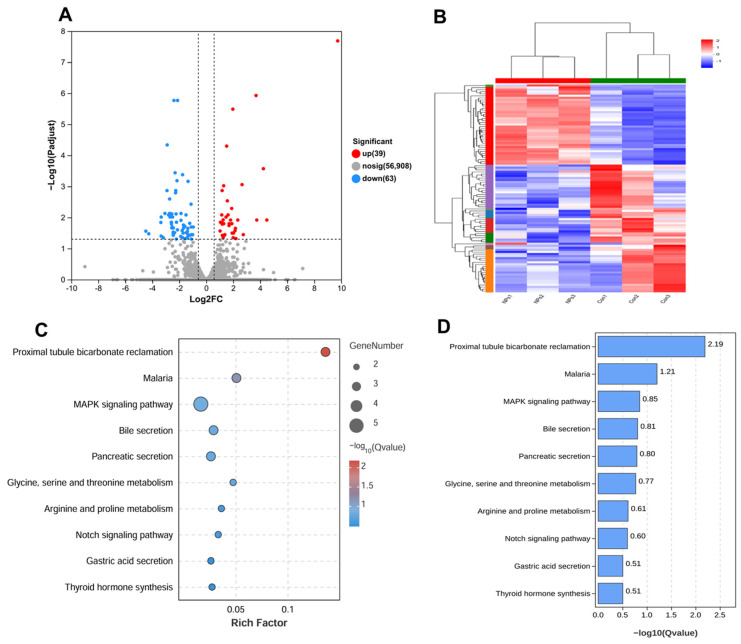
Transcriptomic analyses were performed on the control and 10 mg/kg/d PS-NP groups. (**A**) Volcano plots showing differential gene expression between control and PS-NP-treated groups. Red, blue, and black dots represent DEG upregulated, DEG downregulated, and no differentially expressed genes, respectively. (**B**) Cluster analysis plot of mRNA differentially expressed genes. (**C**) KEGG pathway enrichment analysis bubble plot. (**D**) KEGG pathway enrichment analysis bar graph.

In order to investigate the cause of placental underdevelopment due to PS-NP exposure, we employed transcriptome sequencing to identify differentially expressed genes post-PS-NP exposure in placental tissue. Gene expression differences were examined using DESeq, setting the filtering criteria for differentially expressed genes as |log2FoldChange| > 2 and FDR < 0.05. The results revealed an upregulation of 39 genes and downregulation of 63 genes in the placental tissue post PS-NP exposure, giving a total of 102 differentially expressed genes. A volcano plot was used to illustrate the overall differential distribution and aid in the selection of such genes (Figure 5A). Cluster analysis suggested that genes within each sample with similar expression patterns clustered together, with distinct separation between the two groups, indicating no layered confusion (Figure 5B).

We performed a KEGG pathway enrichment analysis on genes linked with PS-NP exposure using the KEGG database. Based on the results from the KEGG enrichment analysis of the differentially expressed genes, we selected the top 10 most significantly enriched pathways (those with the smallest *p*-value) (Figure 5C,D). The enrichment of differentially expressed genes was most significant in the MAPK signaling pathway. The downregulation of the MAPK signaling pathway is closely linked to embryonic growth and development, suggesting that regulatory factors related to this pathway may be associated with embryonic developmental delay induced by NP exposure. Further bioinformatics results are available in the Supplementary Material, Figure S3. Subsequently, we selected four genes, MAP2K6, Cacng4, Flt1, and Dtx3l, which showed differential expression in the sequencing results, for qRT-PCR validation. The results were consistent with the sequencing data (Supplementary Material, Figure S4).

### 3.4. Effects of PS-NPs on Trophoblast Cells

**Figure 6:**
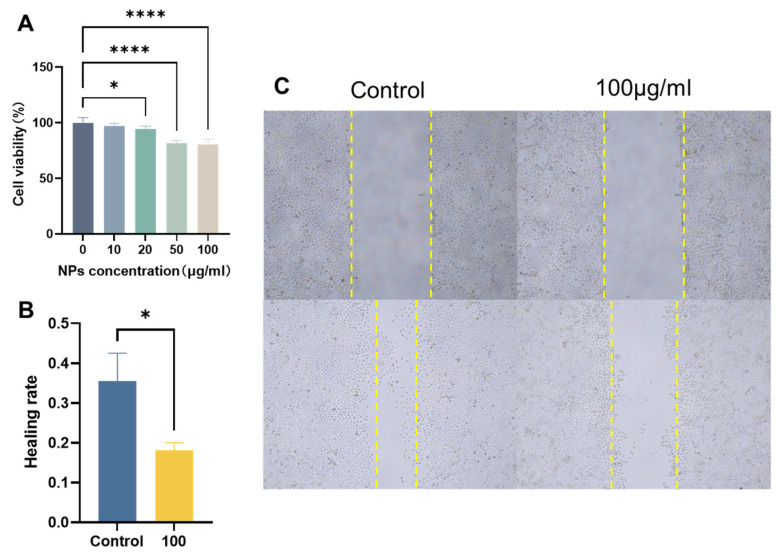
Exposure of PS-NPs inhibits HTR cell viability, migration, and invasion in vitro. (**A**) Twenty-four-hour cell viability of HTR cells exposed to the indicated doses was determined by Cell Counting Kit-8 assay. (**B**) Quantitative results of wound healing assay control and high-dose groups. (**C**) PS-NP exposure reduces the migration distance of HTR cells. Scale bar = 100 μm. Data are presented as the mean ± SD of three independent assays. * *p* < 0.05, **** *p* < 0.0001.

To further corroborate the correlation between the impact of PS-NPs on placental function and trophoblast cells, we exposed HTR cells to PS-NPs. The CCK8 assays demonstrated a significant reduction in HTR cells’ viability, with the high-dose group experiencing an inhibition rate of up to 20% due to PS-NPs (Figure 6A). Moreover, an exposure to 100 μg/L of PS-NPs detrimentally impacted HTR cells’ migration, as evidenced by the scratch tests (Figure 6B,C).

**Figure 7:**
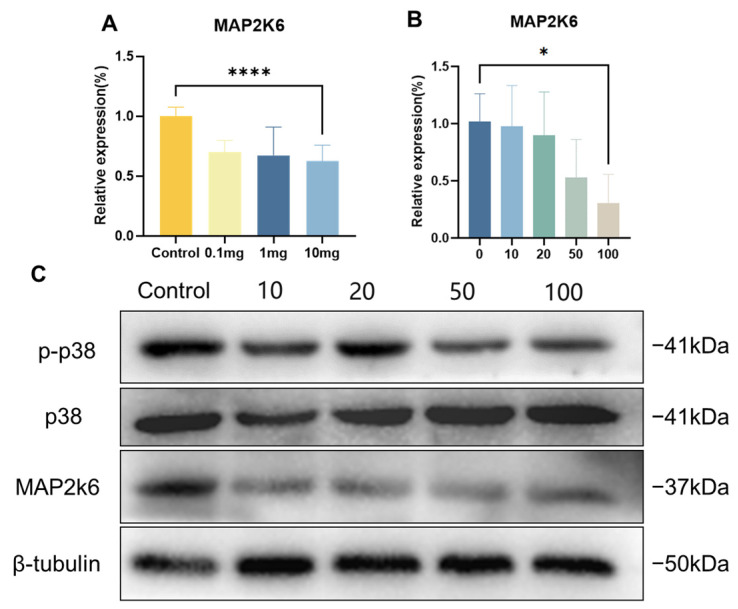
PS-NPs inhibit the expression of p38 MAPK-regulated genes in embryonic growth and development in vivo and in vitro by reducing MAP2K6. (**A**) mRNA levels of MAP2K6 in the placenta of GD18.5 female rats were detected by qRT-PCR. (**B**) Detection of mRNA levels of MAP2K6 by qRT-PCR in HTR cells exposed to the indicated PS-NP doses. (**C**) Protein expression of MAP2K6, p38, and p-p38 in HTR cells exposed to PS-NPs was analyzed by Western blot. Data are presented as the mean ± SD of three independent assays. * *p* < 0.05, **** *p* < 0.0001.

MAP2K6, a vital gene within the MAPK pathway, exhibited reduced expression levels in placental tissues upon exposure to PS-NPs, as discovered through the transcriptome sequencing and qRT-PCR results (Figure 7A). To pinpoint the mechanism underlying the impact of PS-NP exposure on trophoblast cells’ proliferation and migration, we examined the expression levels of MAP2K6. We determined that NP exposure led to diminished mRNA and protein levels of MAP2K6 in the trophoblast cells (Figure 7B). In addition, MAP2K6 is involved in p38 phosphorylation. Normally, an activated MAP2K6, or MKK6, uniquely phosphorylates and activates p38 MAP kinase. The Western blot tests on HTR cells indicated that NP infection reduced the expression of phosphorylated p38 protein, but did not alter the overall p38 expression (Figure 7C).

## 4. Discussion

The widespread detection of nanoplastics has raised concerns about their exposure risks to the environment and human health. In recent years, several studies have demonstrated the toxicity of nanoplastics to mammals [25,26,27,28,29]. Nanoplastics can accumulate in the liver, kidneys, and intestines of mice, causing disruption of energy and lipid metabolism as well as oxidative stress [25]. Exposure to PS-NPs can induce gut microbiota dysbiosis, intestinal barrier dysfunction, and metabolic disorder in mice [27]. Moreover, it has been discovered that exposure to PS-NPs during pregnancy and lactation can modify the function, structure, and cellular makeup of the offspring’s neural stem cells. This leads to neurophysiological and cognitive impairments. In male mice, this exposure also triggers testicular dysgenesis, subsequently influencing their fertility [30,31]. However, studies on the potential harmful effects of nanoplastic exposure during pregnancy on placental embryos are still limited. Therefore, we established a pregnancy exposure model for PS-NPs in ICR mice and screened for differentially expressed genes in placental tissue after exposure to PS-NPs during pregnancy. In addition, we conducted corresponding verifications in the human trophoblast cell line (HTR) and further explored its possible mechanisms.

In mice experiments, exposure to PS-NPs during pregnancy had little effect on the weight, or food and water consumption, of the dams, and did not affect the survival rate or gender ratio of their offspring, which is consistent with the research results of Luo and others [32,33]. However, by weighing and measuring the diameter of the mother mouse’s placenta and embryos, it was found that exposure to PS-NPs during pregnancy reduced the diameter and weight of the placenta. At the same time, pathological sections of the placenta showed that the trophoblastic layer of the treated group was thin, with insufficient invasion of trophoblastic cells, and a large number of immature red cells in the blood vessels. These results all indicate that PS-NPs may break the placental barrier and affect the growth and development of the offspring, and these adverse effects may be related to pathological changes in the placenta. Research indicates that microplastics have now infiltrated the human placenta, with a detection rate as high as 100% in samples taken after 2021 [34]. Furthermore, exposure to PS-NPs can stimulate oxidative stress in placental cells in vitro, increasing the production of ROS, inducing DNA damage, suppressing cell vitality, increasing cell apoptosis, and blocking the cell cycle, and the cytotoxicity of PS-NPs is related to particle size and whether it carries a charge [35,36]. All of these results suggest that exposure to PS-NPs during pregnancy adversely affects embryonic development. 

Based on the impact of prenatal PS-NP exposure on the placenta, we further explored the gene changes in the placenta after prenatal exposure to PS-NPs using RNA-seq technology. Results showed that a total of 102 genes in the placental tissue exhibited different expression after prenatal exposure to PS-NPs, indicating that PS-NP treatment can induce significant changes in the overall gene transcription profile of the placenta. Moreover, KEGG enrichment analysis showed differences in the MAPK signaling pathway, bile secretion, pancreatic secretion, Notch signaling pathway, gastric acid secretion, and thyroid hormone synthesis, among which the MAPK signaling pathway was particularly noteworthy. Therefore, we targeted the p38 MAPK signaling pathway to explore the molecular mechanism of PS-NPs’ toxicity.

In the p38 MAPK signaling pathway, MAP2K6 serves as a crucial regulatory switch in the cellular response to cytokines and growth factors [37,38,39]. Once activated, MAP2K6 phosphorylates p38 MAPK, thereby activating p38. The active form of p38 can then transfer these signals to the downstream targets, which then modulate various cellular activities such as inflammation, cell proliferation, differentiation, and apoptosis. In essence, MAP2K6 serves as a molecular switch to relay the signals to the appropriate response points in the cell when it receives a stimulus from cytokines and growth factors. It is this precise regulation by MAP2K6 that ensures the cells react correctly to these external stimuli [37]. The potential mechanism of the MAP2K6/p38 MAPK axis in cell growth, differentiation, apoptosis, movement, and inflammation has garnered widespread attention. Multiple studies show that a decline in the expression level of MAP2K6 can inhibit the phosphorylation of p38, leading to a reduction in the level of phospho-p38 (p-p38), which in turn causes a reduction in cell proliferation and migratory invasion capabilities. By using transfection to decrease the level of MAP2K6 in human gastric cancer cells, the phosphorylation of p38 can be inhibited, affecting autophagy in GC cells, inducing a G2 phase cell cycle block, and suppressing cell proliferation and migration [40]. The use of MAP2K6 inhibitors can suppress the growth of esophageal cancer cells both in vitro and in vivo [41]. Moreover, the p38 protein plays a pivotal role in embryonic development by regulating the initial differentiation of trophoblast cells and participating in the formation of the placental blood vessels, to the extent that a significant deficiency in p38 phosphorylation can result in fetal death within the uterus [42,43,44]. After exposure to PS-NPs, we found a decrease in the expression of MAP2K6 in the placentas of pregnant ICR mice and a decrease in cell vitality and migration ability in HTR cells. These results suggest that exposure to PS-NPs induces a decrease in the expression of MAP2K6 in the placenta and trophoblastic cells, thus inhibiting the phosphorylation of p38, and affecting the proliferation and migration of trophoblastic cells, leading to inhibition of placental and embryonic growth. Therefore, PS-NPs may affect placental embryonic growth and development by regulating the MAP2K6/p38 MAPK axis.

## 5. Conclusions

**Figure 8:**
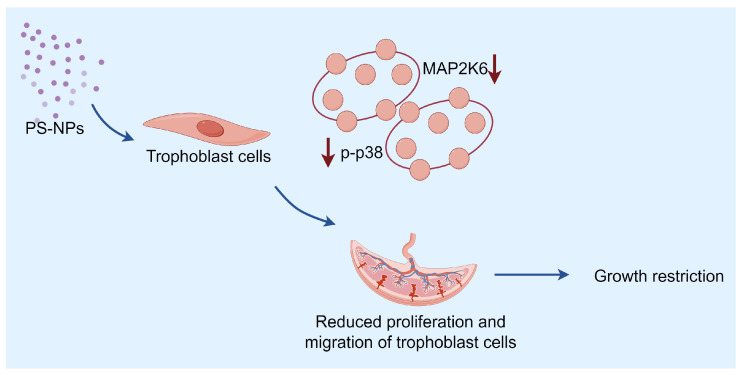
Possible mechanism. PS-NPs may inhibit placental embryo growth and development by decreasing MAP2K6 expression and targeting inhibition of the p38 MAPK pathway, causing embryonic developmental toxicity.

In conclusion, this study investigated the biological effects of PS-NPs on the embryos in the placenta of pregnant mice and HTR cells. The findings suggest that exposure to PS-NPs during pregnancy may not significantly affect the biological responses of the dams, but can notably impact the growth and development of the mice placenta and embryo. The harm inflicted on the placenta by PS-NPs mainly manifests as an inhibition of trophoblast cell proliferation and migration, potentially achieved through the impediment of p38 phosphorylation, regulated by MAP2K6 (Figure 8). This study provides a possible molecular mechanism for understanding the potential embryonic developmental toxicity induced by PS-NPs. The verification results from the HTR cells suggest that there could also be certain impacts on the human trophoblast layer, indicating a need for further exploration in this area.

## Data Availability

Data are contained within the article or Supplementary Material.

## Supplementary Materials

The following supporting information can be downloaded at: https://www.mdpi.com/article/10.3390/toxics12050370/s1, Figure S1: Polystyrene nanoparticle characterization, Figure S2: Biological effects of PS-NP particles on pregnant rats, Figure S3: Differential analysis of placental expression and pathway analysis in PS-NP-exposed pregnant female rats, Figure S4: Validation of qRT-PCR of differentially expressed genes in the placenta of female mice, Table S1: Primers for real time qRT-PCR, Table S2: Antibodies for Western blot.

## Abbreviations

PS-NPs, Polystyrene nanoplastics; TEM images, Transmission Electron Microscope; ICR mice, Institute of Cancer Research mice; HTR, HTR-8/SVneo cells; KEGG, Kyoto Encyclopedia of Genes and Genomes; GO, Gene Ontology; DEGs, Differentially Expressed Genes; PE, polyethylene; PP, polypropylene; PS, polystyrene; PVC, polyvinyl chloride; BCA, Bicinchoninic Acid Assay; SDS, Sodium dodecyl sulfate; PBS, phosphate buffered saline; PVDF, polyvinylidene difluoride; TBST, Tris-buffered saline with Tween 20; qRT-PCR, quantitative reverse transcription polymerase chain reaction; GAPDH, glyceraldehyde-3-phosphate dehydrogenase; MAP2K6, Recombinant Mitogen Activated Protein Kinase Kinase 6; p38, p38 mitogen-activated protein kinase; p-p38, Phospho-p38 mitogen-activated protein kinase; MAPK, mitogen-activated protein kinase; CCK8, Cell Counting Kit-8.
