# Supplementary material for: Inhibitory Impact of Prenatal Exposure to Nano-Polystyrene Particles on the MAP2K6/p38 MAPK Axis Inducing Embryonic Developmental Abnormalities in Mice
Source: Toxics. 2024 May 17;12(5):370. doi: 10.3390/toxics12050370 (PMC11125576; doi:10.3390/toxics12050370)
Supplement: Supplementary file 1 [file toxics-12-00370-s001.zip › toxics-3000615-supplementary.pdf]

Supplementary Material

Figure S1. Polystyrene nanoparticle (PS-NPs) characterisation. (A) Transmission electron micrograph. (B) Particle size map.

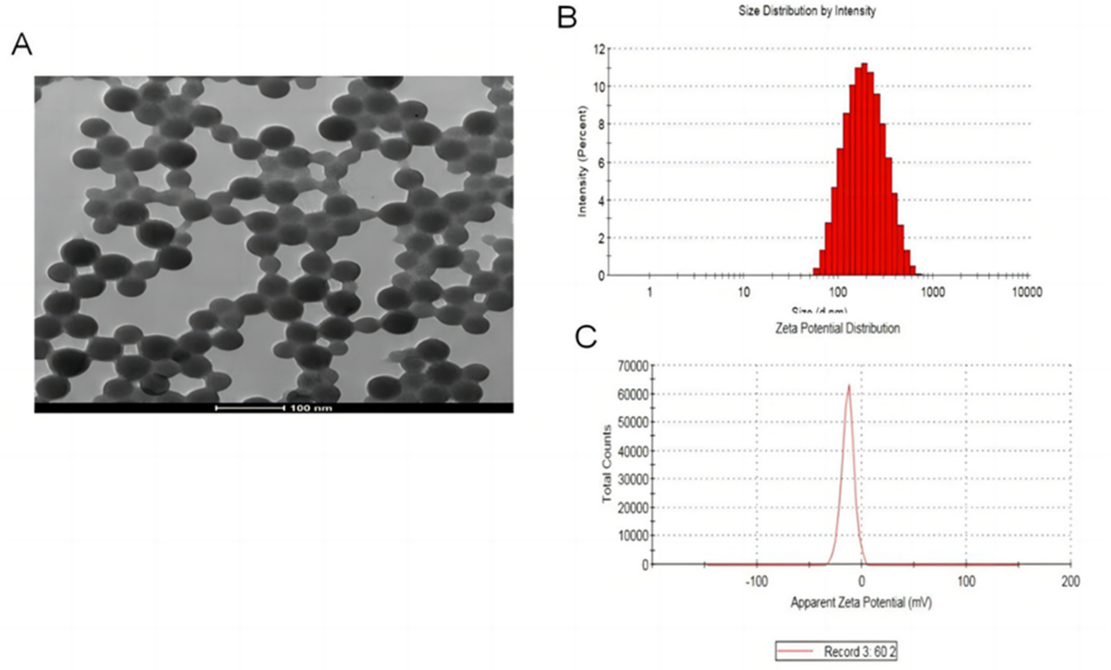

Figure S2. Biological effects of PS-NPs particles on pregnant rats. (A) Body weight of pregnant rats. (B) Trends in body weight of pregnant rats. (C) Food intake of pregnant rats. (D) Water intake of pregnant rats. Data are represented as the mean ± SEM.

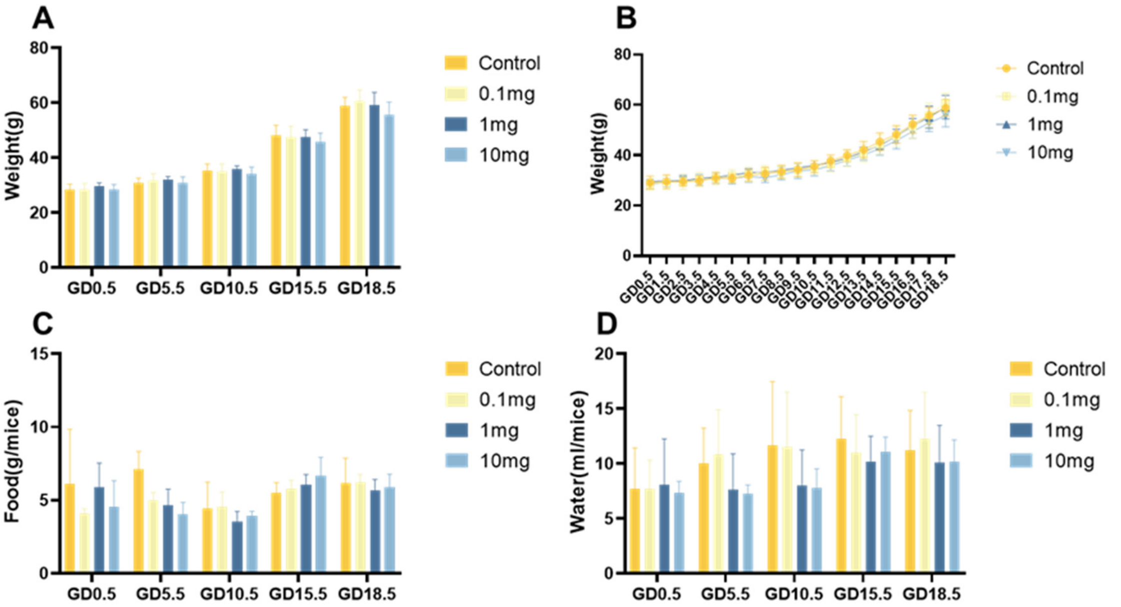

Figure S3. Differential analysis of placental expression and pathway analysis in PS-NPs pregnancy-exposed female rats. (A) MA plot of mRNA differentially expressed genes. (B) GO enrichment analysis bubble plot. (C) GO enrichment analysis histogram. (D) KEGG classification statistics histogram. (E) GO classification statistics bar graph.

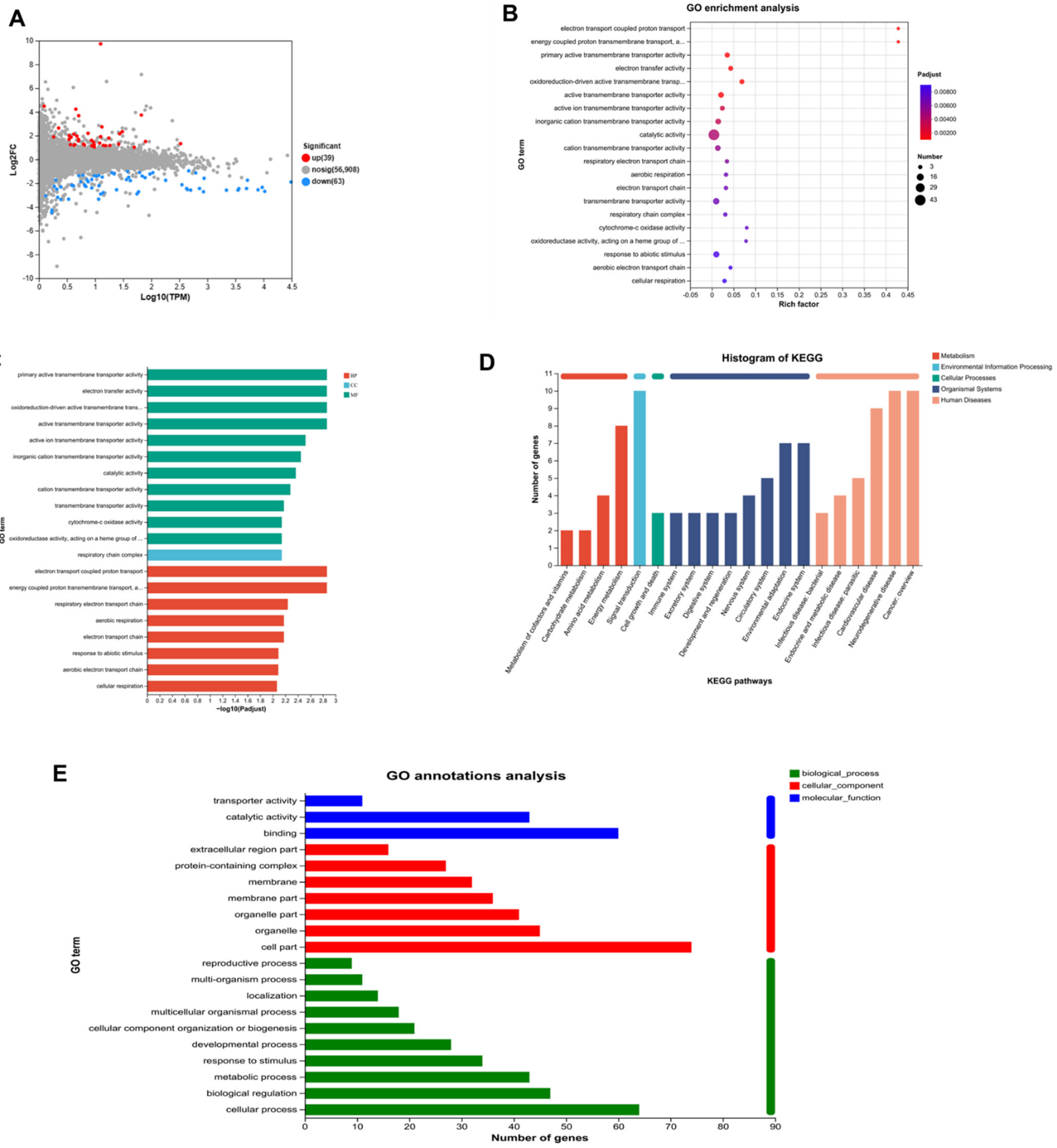

Figure S4. Validation of qRT-PCR of differentially expressed genes in the placenta of female mice. (A) Cacng4. (B) MAP2K6. (C) Flt1. (D) Dtx3l. $^{**}P < 0.01$, $^{****}P < 0.0001$.

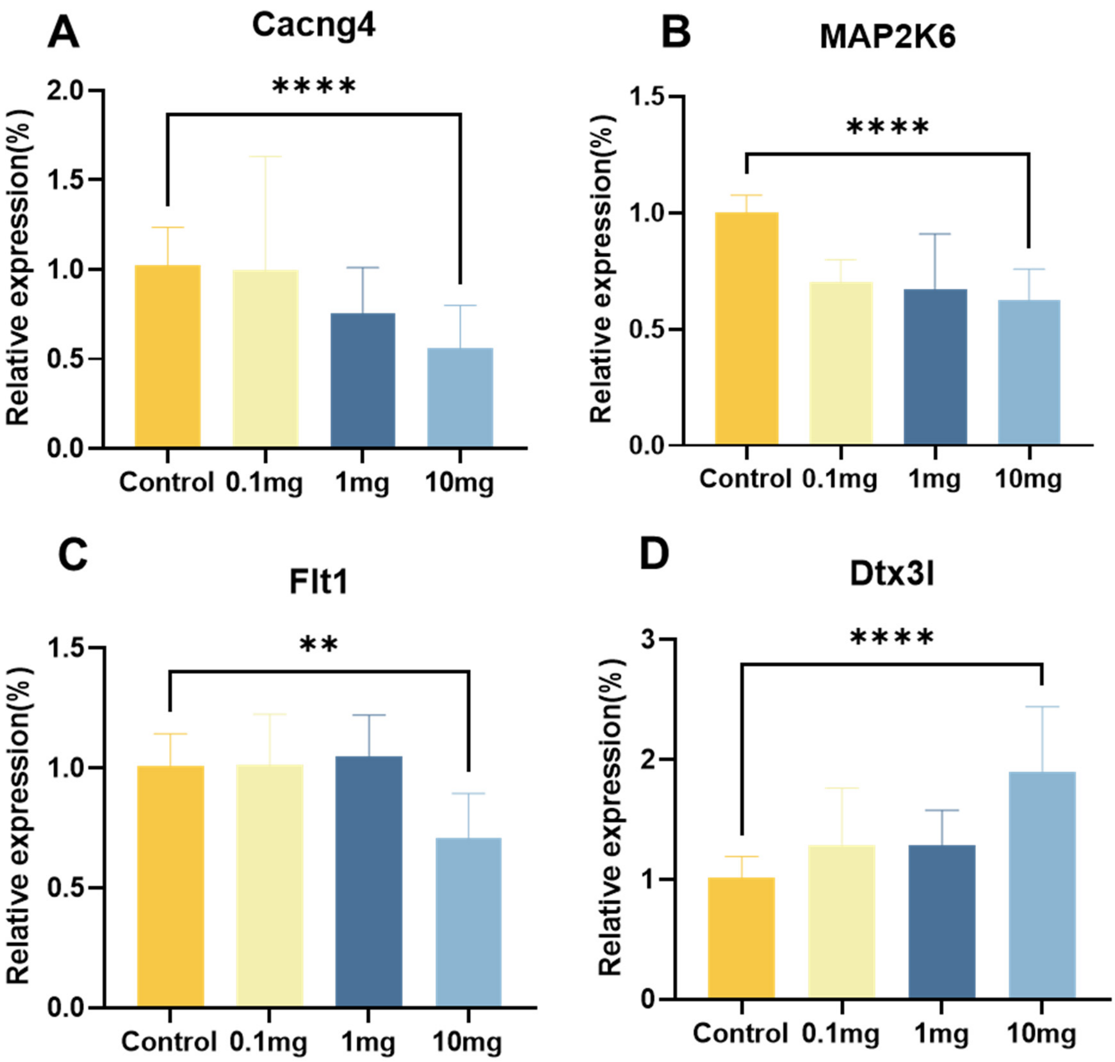

Table S1. Primers for real time qRT-PCR.

Gene		Sequences of primers (5'-3')
GAPDH(MMU)	forward	TGTTTCCTCGTCCCGTAG
	reverse	CAATCTCCACTTTGCCACT
Map2k6(MMU)	forward	ATGTCTCAGTCGAAAGGCAAG
	reverse	TTGGAGTCTAAATCCCGAGGC
Cacng4(MMU)	forward	CCCATCCTCAGCACCATTCT
	reverse	CCCGTGTTGCTGGAAATGTA
Flt1(MMU)	forward	AGGGATAACAGGCAATTCTGC
	reverse	GTGCATCTCTATGAAAGGACTCC
Dtx3l(MMU)	forward	GGTCTCTGGATGAAGCACTT
	reverse	AGAGAGCAGGTCACAGTTCA
GAPDH(HSA)	forward	CAGGAGGCATTGCTGATGAT
	reverse	GAAGGCTGGGGCTCATTT
Map2k6(HSA)	forward	TCAATGCTCTCGGTCAAGTG
	reverse	GTGATGCCCAGACTCCAAAT

Table S2. Antibodies for western blot.

Antibodies	RRID	Source
p38-MAPK(WB1:1000)	AB_2878007	Proteintech,Illinois，USA
Goat anti-rabbit IgG (H+L)(WB1:1000)	AB_2722564	Proteintech,Illinois，USA
Phospho-p38 MAPK(WB1:1000)	AB_2918205	Proteintech,Illinois，USA
Tubulin β polyclonal antibody(WB1:1000)	AB_2797447	Bioworld biotech,Nanjing,China
HRP-conjugated Affinipure Goat Anti-Mouse IgG(H+L)(WB1:1000)	AB_2722565	Proteintech,Illinois，USA
Anti-MAP2K6 (WB1:1000)		Bbi life sciences corporation，
